# Comprehensive Evaluation of Mathematical Models Used in the Thin‐Layer Cold Dried Foods

**DOI:** 10.1002/fsn3.70558

**Published:** 2025-07-01

**Authors:** Aydin Kilic

**Affiliations:** ^1^ Tourism Faculty, Gastronomy and Culinary Art Department Recep Tayyip Erdoğan University Rize Turkey

**Keywords:** cold drying, evaluation, food, mathematical modeling, thin layer

## Abstract

This article focused on the comprehensive evaluation of statistical criteria applied in common mathematical models selected for experimental cold drying data for thin‐layer food drying applications. In this context, Mackerel ( *Trachurus trachurus* ), known as a functional and sensitive food sample with its bioactive content, was selected as the experimental material for drying applications. For this purpose, four experimental groups (G5MM, G10MM, G15MM, G20MM) with different sample thicknesses (5, 10, 15, 20 mm) at 100 g were dried with 6 m/s air flow at 10°C for 24, 22, 20, and 14 h respectively. Twenty‐three common semi‐theoretic and empiric mathematical models were applied to the obtained drying values. For the comprehensive evaluation of the models, non‐linear regression analysis was performed using 13 different statistical criteria such as *r*, *RSS*, *SST*, *SSE*, *R* ^2^, *χ* ^2^, *RMSE*, residuals, *RSSE*, *MBE*, *EF*, *SEE*, and *p*. In this context, in the study where the relevant criteria were applied, for G20MM, Newton Lewis, Midilli‐Küçük, Balbay and Şahin, Page, for G15MM, Henderson & Pabis, Logarithmic (Asymptotic), Binomial, Verma et al., Modified Henderson, Simplified Fick diff., Balbay and Şahin model were concluded to be the most suitable. In addition, for G10MM, Logarithmic (Asymptotic), Demir et al., Binary, Verma et al., Balbay and Şahin, Thompson and Alibas models, and in the G05MM group, Logarithmic (Asymptotic), Demir et al., Binary, Verma et al., Thompson, Balbay‐Şahin and Alibas models were concluded to be the most suitable. According to the results obtained, it has been revealed that using only *r*, *R* ^2^, *χ* ^2^ and *RMSE* equations instead of 13 statistical criteria in the evaluation of mathematical models gives significant and meaningful results.

## Graphical abstract

This study comprehensively evaluates the statistical criteria applied in mathematical models in thin layer drying of foods. As a functional sensitive food, fish was used in different sample thicknesses with high air velocity and cold drying. It was concluded that only *r, R* ^2^, *χ* ^2^ and *RMSE* equations were sufficient in the evaluation of mathematical models. At the same time, it was determined that some models could be selected as common models in all experimental materials.

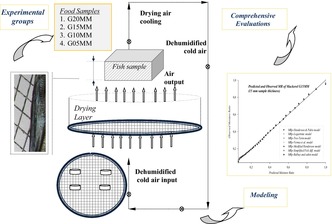

## 1 Introduction

Food drying is one of the oldest food preservation methods. The main purpose of this unit operation applied to eliminate microbial and biochemical quality losses is to reduce the water activity of the product (Zeng et al. 2024). Drying, which has many application differences, is a basic preservative process and is also a part of many food production chains. Free water, also defined as water activity in foods, is one of the main reasons for the reproduction of microorganisms. On the other hand, low‐temperature drying (LTHV) is recommended to prevent quality losses, especially for functional food products such as fish containing bioactive components (Kilic 2009). This basic process, especially applied at low temperatures, can also affect the technological functionality and bioactivity of bioactive ingredients in dried functional foods (Qi et al. 2021; Xiao et al. 2017; Kilic 2022; Kilic 2009). Drying is not always a unit operation applied alone, but is even used together with other preservatives, especially against pathogenic bacteria, in various methods within the scope of hurdle technology (Kilic and Oztan 2013; Zeng et al. 2024). In addition, drying does not contain the harmful side effects of chemical preservatives on human health, and especially cold drying does not have any environmental impact, so it can be defined as an environmentally friendly, healthy, and even sustainable basic process (Kilic 2022, 2009).

It is known that many mathematical models have been applied for the standardization and practical application of the drying process. In this context, there are 67 mathematical models applied in the literature and 28 statistical evaluation criteria applied in the selection of these models (Kucuk, Kilic, et al. 2014; Kucuk, Midilli, et al. 2014). To experimentally discuss the mathematical models and evaluation criteria determined in line with all these explanations, fish, known as one of the most sensitive foods, was selected as experimental material for drying experiments.

In this context, Mackerel ( *Trachurus trachurus* ), selected as the raw material, is known as a commercially valuable fish species with widespread consumption throughout the world (Zarandona et al. 2021). This species, produced by hunting, is widely offered for fresh consumption. On the other hand, the extreme decrease in the price of the product, especially in some periods, reveals the need to process it into other products with higher returns (Zarandona et al. 2021; Kilic 2022). The alternative of processing the product into a durable product during the abundant season, storing it for a while, and then offering it for sale in the appropriate marketing season will provide significant economic gain for the producer and an alternative product opportunity for the consumer during the low season. These new alternative products can be supported by using a safer, sustainable, and environmentally friendly unit operation such as drying (Kilic 2009, 2022). The data obtained from the thin layer cold drying of commercial mackerel fish were evaluated with many different models selected from among the numerous mathematical models available in the literature and were comprehensively evaluated with the criteria selected from among the numerous statistical evaluation criteria available in the literature.

Modeling the drying process of food presents challenges due to the large number of food and environmental variables and unavoidable factors (Trystram 2012; Djekic et al. 2019). It can be said that the models of cold air‐drying as thin layer widely used in industry and literature are semi‐theoretical, theoretical, and empirical commonly (Onwude et al. 2016; Baidhe and Clementson 2024; Ertekin and Firat 2017; Turan and Firatligil 2019). On the other hand, the models applied consider the strength that resists the passage of water vapor between the food material and the surrounding atmosphere (Kucuk, Midilli, et al. 2014; Baidhe and Clementson 2024; Akpinar 2006). Fick's second law, a theoretical model, is a law widely used, especially in thin films drying applications of foods (Kilic 2017; Kucuk, Kilic, et al. 2014). Theoretical models derived from Fick's second law without the need for experimental data, although they explain the drying process, may lead to some deficiencies and errors in terms of practical applications.

Nowadays, developed mathematical models are used to redesign various drying systems specifically for the product used and to determine the most ideal drying parameters for the relevant product. These models are also used to correctly determine the heat and mass transfer characteristics (Kilic 2017, 2020; Kucuk, Midilli, et al. 2014). The selected models are also applied to determine the drying kinetics of heat‐sensitive functional foods with bioactive content (Chin et al. 2008). In summary, modeling consists of simplified and detailed mathematical equations to define the drying system and parameters.

This utilizes detailed mathematical models, variable parameters contained in the raw material, heat and mass transfer equations. These equations can be defined as a non‐linear, partial differential equation system (Sorokova et al. 2022). The specific best models determined for each product are required for the selection of drying systems and parameters suitable for the specific product in industrial applications (Kilic 2017; Kucuk, Midilli, et al. 2014; Baidhe and Clementson 2024). This situation is of key importance in understanding whether the manufacturer is making high quality and economical production and whether the thin film drying system used is efficient (Kilic 2017; Kucuk, Midilli, et al. 2014).

Determining the most suitable equation to be applied for thin layer cold drying of foods is an important scientific research stage in terms of scientific studies, engineering education, and food industry practices. In this context, to determine the most suitable models, 23 different theoretic, empiric, semi‐theoretic, and theoretical mathematical equations widely used were subjected to nonlinear regression analysis on experimental data atmosphere (Kidane et al. 2025; Kucuk, Midilli, et al. 2014; Baidhe and Clementson 2024; Akpinar 2006; Buzrul 2022). Comprehensive and in‐depth comparisons were made according to the obtained coefficients of determination (*r*, *RSS*, *SSE*, *R* ^2^, *χ* ^2^, *RMSE*, residuals, *EF*, *SEE, RSSE*, *MBE*, *p*). While the maximum value obtained from some analysis criteria such as *R* ^2^ and *r* value is decisive in determining the most appropriate mathematical models, the minimum value obtained from some criteria may be decisive for the most appropriate model, such as *χ* ^2^ and *RMSE* (Ertekin and Firat 2017; Ruiz‐López and Herman‐Lara 2009).

Akpinar et al. (2003) used *r*, *χ* ^2^ and *RMSE* criteria in a similar way for statistical evaluation in the thin layer drying of red pepper as plant‐based food. In this context, he determined the values as 0.9987, 0.000332, and 0.0174, respectively. Akpinar (2006) also studied the determination of a suitable thin layer drying‐curve model for 13 models in some vegetables and fruits. There are plant and animal products in the literature where thin layer drying is applied (Kilic 2009, 2017, 2020; Buzrul 2022). When these studies, especially those where low temperature and high‐speed drying are applied, are examined, there are non‐linear similarities in the drying properties of both plant tissues containing plant cell walls and animal cell tissues containing support tissues such as connective tissue and adipose tissue. It is also understood that there are similarities in the selected models and evaluation criteria. In the literature study, it was determined that *R* ^2^, *χ* ^2^ and *RMSE* criteria were used in the selection of the most suitable model in the mathematical modeling of thin layer drying of fig, olive, tomato, yarrow, coriander, marshmallow, alga, peppers, rough rice, carrot, coffee, thymus, chilis, turmeric, orange peels, tomato, potato, fruit, ginger, apples, apricots, Cuminum as animal sources food (Basunia and Abe 2001; Cerezal‐Mezquita and Bugueno‐Munoz 2022; Deeto et al. 2018; El‐Sebaii and Shalaby 2013; Ekka et al. 2021; Mellalou et al. 2023; Umayal Sundari and Veeramanipriya 2022; Zomorodian and Moradi 2010; Ghatrehsamani et al. 2012; Boughali et al. 2009; Amanlou et al. 2015; Stegou‐Sagia and Fragkou 2018; Azaizia et al. 2020; Gupta et al. 2020; Karthikeyan and Murugavelh 2018; Lakshmi et al. 2018; Mahapatra and Tripathy 2018; Murugavelh et al. 2023; Onyenwigwe et al. 2023; Poonia et al. 2018; Suherman et al. 2021; Kidane et al. 2025; Onwude et al. 2016).

This article aims to evaluate the common statistical criteria used in the analysis of mathematical models widely used in the literature and to select the most appropriate model and evaluation criteria. In this context, the thin layer drying behavior on fish ( *T. trachurus* ) selected as raw material was analyzed using the common thin layer LTHV method, comprehensive mathematical modeling, and comprehensive statistical criteria for the selection of the best model. Together with these experimental results, comparative models and statistical evaluation criteria with thin layer drying studies applied to different plant and animal origin food products in the literature aimed to present a new approach with more practical modeling and practical evaluation and to suggest practical applications for all industrial foods (Erbay and Icier 2010; Kidane et al. 2025).

## 2 Material and Methods

### 2.1 Mathematical Modeling

Although there are many semi‐theoretical and/or empirical mathematical models in the literature, the most widely used 23 models (Equations (5), (6), (7), (8), (9), (10), (11), (12), (13), (14), (15), (16), (17), (18), (19), (20), (21), (22), (23), (24), (25), (26), (27)) were determined and applied to the experimentally obtained data. While 67 models and 28 evaluation criteria were evaluated in the compilation study conducted by Küçük et al. we only considered the most common 23 models and 13 evaluation criteria in this study. In this regard, the values obtained from the mathematical equations were comprehensively analyzed by applying non‐linear regression with the Statistica 10.0 PC program and applying a total of 13 evaluation equations (Equations (28), (30), (31), (32), (33), (34), (35), (36), (37), (38), (39), (40)), namely, the correlation coefficient (*r*), the standard error of estimate (*SEE*), the reduced sum square error (*RSSE*), the coefficient of determination (*R* ^2^), residual sum of squares (*RSS*), the error (residual) sum of squares (*SSE*), the reduced chi‐square (*χ* ^2^), modeling efficiency (*EF*), the mean bias error (*MBE*), the mean relative percentage error (*P*), the root mean square error (*RMSE*), residuals, and adjusted (${\bar{R}}^{2}$). In this context, the estimated moisture rates for each mathematical model were determined and graphs were drawn based on the changes in experimental drying data over time. Sigma Plot graphic drawing and data analysis software were used in drawing the graphs. The obtained data and constants were evaluated with statistical evaluation equations (Kucuk, Kilic, et al. 2014; Kucuk, Midilli, et al. 2014; Onwude et al. 2016; Buzrul 2022).

To determine the most compatible equation, correlation coefficients *r*, *R* ^2^, *χ* ^2^ and *RMSE* values were accepted as references. The determination of the most appropriate mathematical models, the maximum values obtained from *R* ^2^, *r*, and the minimum of *χ* ^2^, *RMSE* are taken as the basis for the significant variations. To make a comprehensive evaluation, a comparison was made within the scope of 23 models and 13 evaluation criteria based on the review study conducted by Kucuk, Midilli, et al. (2014) and the study conducted by Kucuk, Kilic, et al. (2014) in which common models were determined. Although 13 criteria were used in the comprehensive comparisons, the correlation coefficients *r*, *R* ^2^, *χ* ^2^ and *RMSE* values were accepted as references to determine the most compatible equation. In determining the most suitable mathematical models, the maximum values obtained from *R* ^2^, *r*, and the minimum values of *χ* ^2^, *RMSE* were taken as the basis for significant changes. In the selection of these criteria, significance tests were made on the differences between different variants, and the data obtained were also compared with literature information (Akpinar 2006; Kucuk, Midilli, et al. 2014; Hmazi et al. 2024; Omolola et al. 2014; Erbay and Icier 2010).

After drying, horse mackerel meat undergoes color, structural, and biochemical changes as a result of moisture loss. In this sense, the mass shrinkage (*S*_mr_) for the fish ( *T. trachurus* ) was investigated with Equation (1) (Cruess 1958; Kilic 2017).

(1) $$S_{\mathrm{mr}}=\frac{M_{t}\left(t\right)}{M_{0}\left(t=0\right)}$$

In the formula, *S*_mr_ presents the mass shrinkage of Mackerel; here, *M* is the loss in drying; *M*_0_ is the sample weight for *t* = 0; *M*_*e*_ is the balance weight value, and *M*_*t*_ is the sample weight at any time (Kilic 2017). Horse mackerel fish weight losses of the sample were determined by Equation (2) to investigate the cold air thin layer drying characteristics applied depending on different sample thicknesses (Kilic 2017). The moisture ratio (*MR*) of the Mackerel was detected using the Equation (2) below.

(2) $$\mathrm{MR}=\frac{M_{t}-M_{e}}{M_{0}-M_{e}}$$

where *M*_0_ is the material weight for *t* = 0; the material weight is *M*_*e*_ at the equilibrium time of the sample and *M*_*t*_ is the sample mass at *t* time (Kilic 2017; Ortiz‐Jerez et al. 2024).

The drying rate of *T. trachurus* with cold drying application detected with Equation (3).

(3) $$\frac{dM}{dt}=\frac{M_{t+\Delta t}-M_{t}}{\Delta t}$$

The d*M/*d*t* in the equation represents the cold drying speed of the horse mackerel measured at a certain moment, *M*_*t*_ and *M*_*t*_ + ∆*t* represents the water in the product at *t = t +* ∆*t* (Kilic 2017).

The decrease in dew point in horse mackerel due to cold drying was determined using Equation (4) (Kilic 2017). The amount of water in the product was measured during the cold drying period.

(4) $$W=\frac{M_{t}-M_{e}}{M_{0}}\times 100$$

In the Equation (4), *W* represents the moisture in the horse mackerel sample; *M*_0_ represents the moisture of the horse mackerel at *t* = 0; Me represents the sample weight at the equilibrium time and Mt represents the weight value determined at t time (*Kilic* 2017).

Low temperature high velocity (LTHV) drying values determined in experimental groups of horse mackerel with different sample thicknesses were analyzed by performing nonlinear regression analysis with the Statistica 10.0 package program. The semi‐theoretical 23 or empirical mathematical models commonly used in the analyses were determined. A comprehensive analysis was performed by applying *R* ^2^, *χ* ^2^, *P*, *RSS*, *SST*, *RSSE*, *MBE*, *RMSE*, residuals, *EF*, *SEE*, *r*, and *SSE* statistical criteria to the obtained data. The maximum values obtained from *R* ^2^, *r*, and the minimum values of *χ* ^2^, *RMSE* were taken as the basis for criteria, which were decisive in selecting the best models (Hmazi et al. 2024; Akpinar et al. 2003; Turan and Firatligil 2019).

The selected common mathematical models are given as Equations ((5), (6), (7), (8), (9), (10), (11), (12), (13), (14), (15), (16), (17), (18), (19), (20), (21), (22), (23), (24), (25), (26), (27)) (Kilic 2017, 2020; Kucuk, Midilli, et al. 2014).

(5) $$t=a\ln\left(\mathrm{MR}\right)+b{\left(\ln\left(\mathrm{MR}\right)\right)}^{2}\;\text{Thompson}$$

(6) $$\mathrm{MR}=1+\mathrm{at}+bt^{2}\;\text{Wang and Singh}$$

(7)

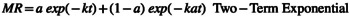

(8) $$\begin{array}{c} \mathrm{MR}=a\;\text{exp}\left(-\mathrm{kt}\right)+\left(1-a\right)\;\exp\left(-\mathrm{gt}\right)\;\text{Verma}\;\mathrm{et}\;\mathrm{al}.\\ \left(\text{Modified}\;\mathrm{Two}-\text{Term Exponential}\right) \end{array}$$

(9)

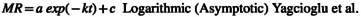



(10)

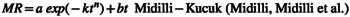

(11) $$\mathrm{MR}=a\;\text{exp}{\left(-\mathrm{kt}\right)}^{n}+b\;\text{Demir}\;\mathrm{et}\;\mathrm{al}.$$

(12)

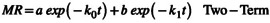

(13) $$\begin{array}{c} \mathrm{MR}=a\;\text{exp}\left(-\mathrm{kt}\right)+\left(1-a\right)\;\exp\left(-\mathrm{kbt}\right)\\ \text{Approximation of Diffusion}\;\left(\text{Diffusion approach}\right) \end{array}$$

(14) $$\begin{array}{c} \mathrm{MR}=a\;\text{exp}\left(-\mathrm{kt}\right)+b\;\text{exp}\left(-\mathrm{gt}\right)+c\;\text{exp}\left(-\mathrm{ht}\right)\\ \text{Modified Henderson and Pabis} \end{array}$$

(15) $$\mathrm{MR}=\exp\left(-\mathrm{kt}\right)\;\text{Newton}\;\left(\text{Lewis},\text{Exponential},\text{Single exponential}\right)$$

(16) $$\mathrm{MR}=\exp\left(-kt^{n}\right)\;\text{Page}$$

(17) $$\mathrm{MR}=\exp\left(-{\left(\mathrm{kt}\right)}^{n}\right)\;\text{Modified Page}$$

(18) $$\mathrm{MR}=\exp\left(-{\left(\frac{t}{a}\right)}^{b}\right)\;\text{Weibull}$$

(19) $$\mathrm{MR}=\exp\left(-\frac{k_{1}t}{1+k_{2}t}\right)\;\text{Aghbashlo}\;\mathrm{et}\;\mathrm{al}.$$

(20)

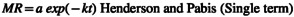



(21)

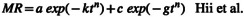

(22) $$\mathrm{MR}=a+\mathrm{bt}+ct^{2}\;\text{Parabolic}$$

(23)

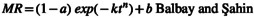



(24)

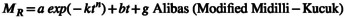



(25)

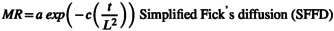

(26) $$\mathrm{MR}=\exp\left({\left(-\mathrm{kt}\right)}^{n}\right)\;\text{Modified Page}-I$$

(27) $$\mathrm{MR}=\exp\left(-c{\left(\frac{t}{L^{2}}\right)}^{n}\right)\;\text{Modified Page}-\mathrm{II}$$

The selection of the most suitable mathematic models analyzed by using statistical Equation (13) different evaluation criteria below as Equations ((28), (30), (31), (32), (33), (34), (35), (36), (37), (38), (39), (40)) (Kilic 2017, 2020; Kucuk, Midilli, et al. 2014).

(28) $$\mathrm{RSS}=\sum_{i=1}^{N}{\left(MR_{\exp,i}-MR_{\mathrm{pre},i}\right)}^{2}\;\text{Residual}\;\mathrm{sum}\;\text{of squares}$$

(29) $$R^{2}=\frac{\mathrm{SSR}}{\mathrm{SST}}=1-\frac{\mathrm{SSE}}{\mathrm{SST}}\;\text{Coefficient of determination}$$

(30) $${\bar{R}}^{2}=1-\left(1-R^{2}\right)\frac{N-1}{N-k-1}\;\text{Adjusted}\;{\bar{R}}^{2}$$

(31) $$\text{RMSE}=\sqrt{\frac{\sum_{i=1}^{N}{\left(MR_{\mathrm{pre},i}-MR_{\exp,i}\right)}^{2}}{N}}\;\text{Root means square error}$$

(32) $$\text{residuals}=\sum_{i=1}^{N}\left(MR_{\exp,i}-MR_{\mathrm{pre},i}\right)\;\text{Residuals}$$

(33) $$\mathrm{EF}=\frac{\sum_{i=1}^{N}{\left(MR_{\exp,i}-MR_{\exp,\mathrm{ave}}\right)}^{2}-\sum_{i=1}^{N}{\left(MR_{\mathrm{pre},i}-MR_{\exp,i}\right)}^{2}}{\sum_{i=1}^{N}{\left(MR_{\exp,i}-MR_{\exp,\mathrm{ave}}\right)}^{2}}\;\text{Modeling efficiency}$$

(34) $$\chi^{2}=\frac{\sum_{i=1}^{N}{\left(MR_{\exp,i}-MR_{\mathrm{pre},i}\right)}^{2}}{N-n}\;\text{Reduced}\;\mathrm{chi}-\text{square}$$

(35) $$\mathrm{SEE}=\sqrt{\frac{\sum_{i=1}^{N}{\left(MR_{\exp,i}-MR_{\mathrm{cal},i}\right)}^{2}}{N-n_{p}}}\;\text{Standard error of estimate}$$

(36) $$\mathrm{MBE}=\frac{\sum_{i=1}^{N}\left(MR_{\mathrm{pre},i}-MR_{\exp,i}\right)}{N}\;\text{Mean bias error}$$

(37) $$P=\frac{100}{N}\sum_{i=1}^{N}\frac{\left|MR_{\exp,i}-MR_{\mathrm{pre},i}\right|}{MR_{\exp,i}}\;\text{the mean relative percentage error}$$

(38) $$\mathrm{SSE}=\sum_{i=1}^{N}{\left(MR_{\exp,i}-MR_{\mathrm{pre},i}\right)}^{2}\;\text{Error}\;\left(\text{residual}\right)\;\mathrm{sum}\;\text{of squares}$$

(39) $$\text{RSSE}=\frac{\sum_{i=1}^{N}{\left(MR_{\exp,i}-MR_{\mathrm{cal},i}\right)}^{2}}{N}\;\text{Reduced}\;\mathrm{sum}\;\text{square error}$$

(40) $$r=\frac{N\sum_{i=1}^{N}\left(MR_{\mathrm{pre},i}\right)\left(MR_{\exp,i}\right)-\left(\sum_{i=1}^{N}MR_{\mathrm{pre},i}\right)\left(\sum_{i=1}^{N}MR_{\exp,i}\right)}{\sqrt{\left(N\sum_{i=1}^{N}MR_{\mathrm{pre},i}^{2}-{\left(\sum_{i=1}^{N}MR_{\mathrm{pre},i}\right)}^{2}\right)\left(N\sum_{i=1}^{N}MR_{\exp,i}^{2}-{\left(\sum_{i=1}^{N}MR_{\exp,i}\right)}^{2}\right)}}\;\text{Correlation coefficient}$$

Twenty‐three empirical, semi‐theoretic and theoretic mathematical equations (Equations (5), (6), (7), (8), (9), (10), (11), (12), (13), (14), (15), (16), (17), (18), (19), (20), (21), (22), (23), (24), (25), (26), (27)) widely used in the literature were applied to the experimentally obtained data (Kucuk, Kilic, et al. 2014; Kucuk, Midilli, et al. 2014; Kilic 2017). During the processes, the estimated moisture rates for each mathematical model were determined and graphs were drawn based on the changes in experimental drying data depending on time. Sigma Plot graphic drawing and data analysis software were used in drawing the graphs. The obtained data and constants were evaluated with statistical evaluation equations. The values obtained from the mathematical equations were comprehensively analyzed by applying regression as a non‐linear with the Statistica 10.0 PC software and applying a total of 13 evaluation equations (Equations (28), (30), (31), (32), (33), (34), (35), (36), (37), (38), (39), (40)), the correlation coefficient (*r*), the standard error of estimate (*SEE*), the reduced sum square error (*RSSE*), the coefficient of determination (*R* ^2^), residual sum of squares (*RSS*), the error (residual) sum of squares (*SSE*), the reduced chi‐square (*χ* ^*2*^), modeling efficiency (*EF*), the mean bias error (*MBE*), the mean relative percentage error (*P*), the root mean square error (*RMSE*), residuals, and adjusted *R* ^2^ (${\bar{R}}^{2}$).

To determine the most compatible equation, correlation coefficients *r*, *R* ^2^, *χ* ^2^ and *RMSE* values were taken as basis. In the selection of the most appropriate mathematical models, the maximum values obtained from *r*, *R* ^2^ and the minimum values of *χ* ^2^, *RMSE* were taken as basis (Kucuk, Kilic, et al. 2014; Kucuk, Midilli, et al. 2014; Kilic 2017).

### 2.2 Experimental Design and Procedure

The special tower type dryer system designed for low temperature drying of *T. trachurus* fish can be defined as a tower type drying system with a diameter of 30 cm, which can cool and heat simultaneously and is supported by programmable software. The closed‐circuit cold dryer cabin was a digital system with online remote controllable smart features where the required parameters such as air speed, drying air temperature, and water content were automatically measured and recorded.

**FIGURE 1:**
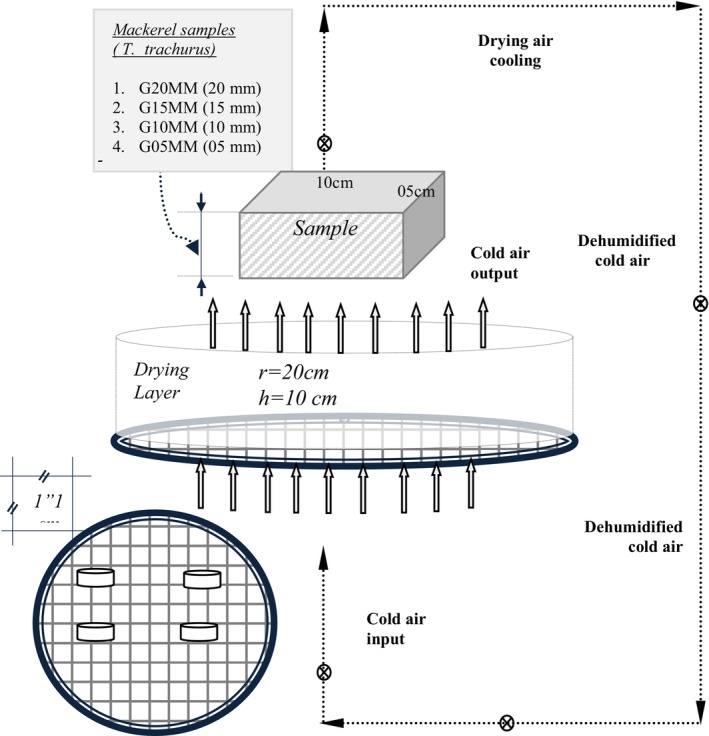
A detailed design of cold air assisted thin layer drying room and sample preparation.

During the cold drying of horse mackerel, the samples were weighed automatically with a digital scale (0.00 ± 0.3 g) mounted on the system. The system was equipped with an anemometer for air speed control, humidity and temperature measuring devices that automatically control the system by recording humidity and temperature (TA2; 0.00 ± 0.3 g), and precision (0.003 ± 0.1) sensors integrated into the system (*Kilic* 2017). Figure 1 presents a detailed design of cold air assisted with thin layer drying room and sample preparation.

### 2.3 Sample Preparation

Moisture analysis was performed to determine the equilibrium moisture content of the samples according to the method recommended by AOAC (1995 Official Analysis Chemists) in the raw material before drying and in each experimental group after drying. In this context, 2 g of fish meat sample was dried in the oven (±105°C) until it reached weight, and the moisture content was determined (% wb). The results obtained were initially determined as approximately 64.8% in the raw material. Then, fish fillet samples with 20, 15, 10, and 5 mm wall thickness, 10 cm width, and 5 cm width were prepared at 10°C ± 1°C cold drying to investigate the cold drying properties of the raw material. Fish samples were prepared in the same order as *G20MM* (20, 100, 50 mm dimensions), *G15MM* (15, 100, 50 mm dimensions), *G10MM* (10, 100, 50 mm dimensions), *G05MM* (05, 100, 50 mm dimensions) and placed in a cold drying cabinet. The dimensions of the samples were determined by preliminary tests to be at a density where the samples would not partially or fully fluidize under the influence of high‐speed drying air velocity (6 m/s), and the experimental samples with the smallest volume were created according to these preliminary test results.

Cold air‐drying of *T. trachurus* was obtained alive from a fisherman who had just caught it from the Black Sea. In the study where fish with an average weight of ~15 cm and 50 g were used according to the measurements, the product that was turned into a single piece fillet by removing the bones, fins, head, and skin was used in the experiments. To reveal the behavior of the single layer and cold air‐drying characteristic of the raw material (*T. trachurus*) whose average moisture content was determined at the beginning, at different muscle thicknesses, it was carried out using a closed tower type drying cabinet at 10°C at a cold air speed of 6 m s^−1^ ± 0.3. The *a*_w_ value was determined with an Aqua Lab Model cx2 device with a sensitivity of ±0.003 for *T. trachurus* .

**FIGURE 2:**
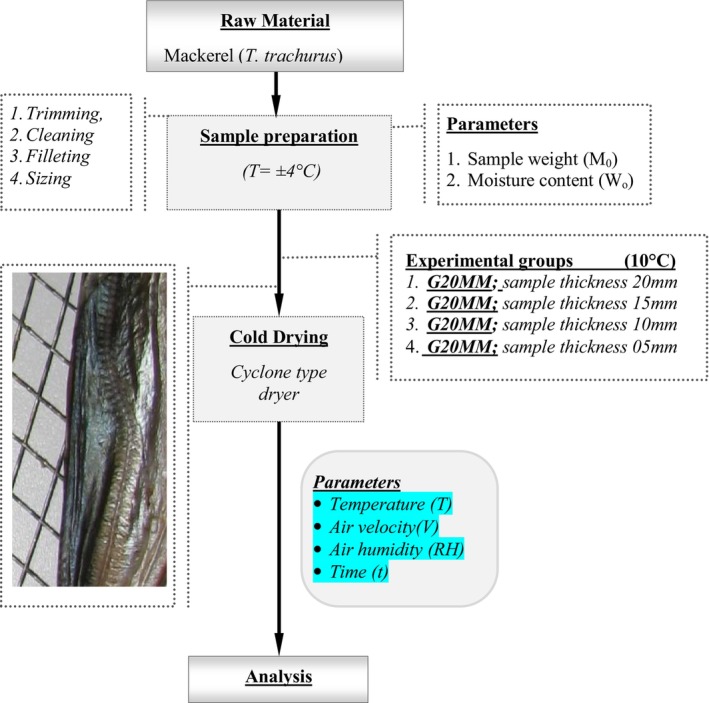
The process steps applied in the cold drying of Mackerel.

Figure 2 shows the process steps applied in the cold drying of Mackerel.

Raw material moisture content in horse mackerel fish was determined to be approximately 64.8% (wb). In the product, which was filleted by removing the bones, fins, and skin, all groups were prepared as 5 × 10 cm slices, while the same groups were labeled by dividing into different sample groups with 5 thicknesses. The 5 mm wall thickness group was labeled as *G05MM*, the 10 mm thick group as *G10MM*, the 15 mm thick group as *G15MM*, and the 20 mm thick group as *G20MM*. Samples were placed on stainless round sieve trays with 1 cm mesh opening for the drying process. During the cold drying processes, relative humidity, temperature, air velocity, and sample weight were recorded and digitally recorded at all environmental, system, and control points determined in the dryer cabin. The average environmental *Rh* value recorded was between 46% and 53%. During the procedures, dry and wet bulb values were determined. All parameters were monitored by determining the cold air inlet and outlet as a control point every half hour. It is known that the drying speed varies depending on the temperature; in this context, the air flow rate applied in cold drying was applied as a high value of 6 m/s to decrease the negative aspect of cold drying to the drying velocity (Putra and Ajiwiguna 2017; Kilic 2009, 2017).

## 3 Results and Discussion

The study is a new application that will reveal the change in the thickness of a variable‐thickness horse mackerel meat sample, unlike previous applications, in which the drying process using cold air (*LTHV*) is applied in a controlled system (Kilic 2017, 2009). While 100°C and 6 m/s cold air speed were selected as low temperature in all samples, there were differences in drying times due to different sample thicknesses. Parameters such as sample weight, humidity, shrinkage, and temperature were continuously recorded during drying. Horse mackerel cold drying parameters and uncertainties are given in Table 1.

**TABLE 1:** The uncertainties of measured parameters of cold drying for G20MM, G15MM, G10MM, and G05MM.

Cold drying parameters	The cold drying parameters for the experimental groups					
	G20MM	G15MM	G10MM	G05MM	Comments	Unit
Sample thickness	20	15	10	5	± 2	mm
Sample length	100	100	100	100	± 3	mm
Wide of the sample	50	50	50	50	± 2	mm
Total drying time	24	22	20	14	—	h
Cold air drying temperature	10	10	10	10	±1	°C
LTHV air drying velocity	6	6	6	6	±0.5	m/s
Cold air inlet temperature	10	10	10	10	±0.3	°C
Air outlet temperature	09	09	09	09	±0.5	°C
The relative humidity of cold air input	37.80	48.30	42.13	41.79	±0.3	%
The relative humidity of cold air output	38.02	48.83	42.32	41.93	±0.3	%
Initial weight	100	100	100	100	±0.4	g
Final weight	50.3	49.8	49.8	50.1	—	g
Initial moisture content (wb)	73	77	75	71	±0.14	%

It is understood from the literature research that there is no study conducted in this context on horse mackerel fish, which is one of the most common commercial fish species and is consumed by a wide range of people and is also known as the dry bean of the seas among the public.

### 3.1 Thin‐Layer Cold Drying Modeling and Evaluation of Mackerel

The cold drying method applied in the study is defined in literature as a new drying method with the symbol *LTHV*. Kilic (2009) states that the *LTHV* drying method gives the most important values in terms of product quality at 4°C, and when drying performance and quality are considered together, it is thought that optimum drying and quality conditions can be provided with 10°C thin and single layer drying. Although the data obtained show that drying at 4°C gives good results in terms of food safety, when considered together with the drying performance, it shows that the optimum parameters can be at 10°C and 7 m/s air speed. In this context, the drying temperature selected in the experiments was determined as 10°C. It has been shown that 7 m/s air speed causes fluidization in the products during the drying process. Therefore, it was concluded that the optimum conditions in the preliminary trials could be the parameters in Table 1. This method is known as an environmentally friendly drying application especially for sensitive functional foods with bioactive content. When determining the fillet thickness of the samples, the fish's flesh thickness and fillet dimensions were taken into consideration. When placing the samples on the drying trays, a gap of sample size was left between them to allow airflow.

Since deviations that may occur during drying at 10°C and 6 m/s air velocity will directly affect the experimental data and mathematical modeling results, system design and stability of drying parameters are very important (Kucuk, Kilic, et al. 2014; Kucuk, Midilli, et al. 2014; Kilic 2009). For these reasons, many parameter control points were selected in the LTHV drying system (CP1, CP2 etc.) and drying parameters (*t*, *Rh*, *v*, *w*) were monitored for 24 h, and digital records were recorded. The selected control points (CP) were determined, such as drying air inlet, outlet, the space of drying layer, cooling inlet, and cooling outlet points. In this context, significance tests were carried out, and since the differences within the linear values were not significant, no additional parameters graph is provided. There are some studies like Low temperature high velocity (LTHV) drying where different parameters are applied similarly (Kilic 2020, 2009). Although the obtained data show that drying at 4°C gives good results in terms of food safety, when considered together with the drying performance, it shows that the optimum parameters can be at 10°C and 7 m/s air speed. It has been shown that 7 m/s air speed causes fluidization in the products during the drying process. Therefore, it was concluded that the optimum conditions in the preliminary trials could be the parameters in Table 1 (Kilic 2009, 2020; Akpinar 2006).

At the beginning of the study, Mackerel samples were 100 g. After cold drying of samples, the weight of samples was detected as 50.3 g at *G20MM* for 24 h, 49.8 g at *G15MM* for 22 h, 49.8 g at *G10MM* for 20 h and 50.1 g at *G05MM* for 14 h. These results have similarities with the *LTHV* drying properties of Trout ( *Oncorhynchus mykiss* , Kilic 2017). Kilic (2009) detected that the samples weight declined to 56 g in 23.5 h at 10°C drying temperature similarly (Kilic 2017, 2022). Kilic (2022) also found that the drying weight of *Engraulis encrasicolus* samples decreased from 200 g to 46.7 g at 10°C for 23 h. These results mean that heat and mass transfer decrease in direct proportion to the increase in sample thickness (Zeng et al. 2024). Since the standardization of the chemical properties and drying parameters of the product was provided to a significant extent, no unusual situation was determined; therefore, all the obtained data were evaluated. If there were deficiencies in the stability and measurement of the experimental parameters, then statistical generalization could not be made. Thus, it would have required repeating the experimental application, ensuring the standardization of the parameters at critical control points and re‐obtaining the data.

**FIGURE 3:**
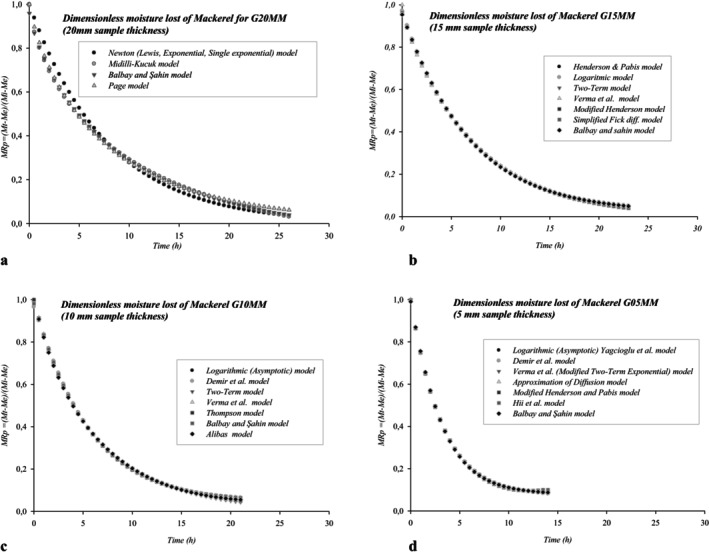
(a–d) The sample moisture comparison on different sample thickness for G20MM, G15MM, G10MM, G05MM using thin layer cold drying at 10°C.

In Figure 3a–d, the dimensionless moisture loss of sample moisture over time depending on different sample thicknesses when thin layer cold drying at 10°C was used in *G20MM*, *G15MM*, *G10MM*, *G05MM* experimental groups is given.

The maximum values obtained in *R* ^2^, *r*, *χ* ^2^ and *RMSE* criteria were decisive in selecting the best models. In the selection of the most appropriate mathematical models, the maximum values obtained from *r*, *R* ^2^ and the minimum values of *χ* ^2^, *RMSE* were taken as a basis (Kucuk, Midilli, et al. 2014; Akpinar 2006; Hmazi et al. 2024; Doymaz 2010; Popescu et al. 2023; Zeng et al. 2024). Hmazi et al. (2024) similarly reported from Oke et al. (2020) *R* ^2^ value of 0.999 and an *RMSE* of 0.00000625 for sweet potato starch drying Similarly, *Akpinar* (2006) reported 0.998 (*r*), 0.00032 (*χ* ^2^), 0.0174 (*RMSE*) for the multiple regression on the coefficients in red pepper.

In this regard, the most suitable 4 models selected for *G20MM* include the Newton Lewis, Midilli‐Kucuk, Balbay, and Şahin‐Page models. The most suitable 7 models selected for *G15MM* include Henderson & Pabis, Logarithmic (Asymptotic), Two‐Term, Verma et al., Modified Henderson, Simplified Fick diff., and Balbay and Şahin models. The most suitable 7 models selected for *G10MM* include Logarithmic (Asymptotic), Demir et al., Two‐Term, Verma et al., Thompson, and Balbay and Şahin Alibas models. The similar 7 models selected for *G05MM* include Logarithmic (Asymptotic), Demir et al., Verma et al., Approximation Diff., Modified Henderson and pubis, Hii et al., and Balbay and Şahin models.

**FIGURE 4:**
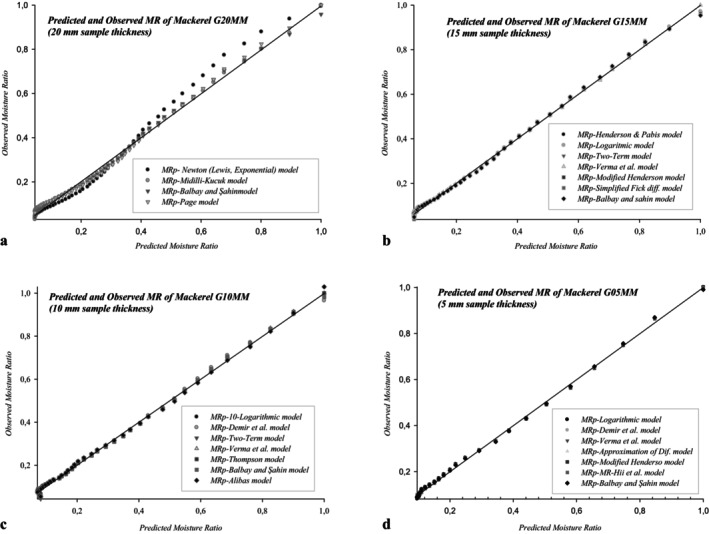
(a–d) The comparison of predicted and experimental MR according to the moisture ratio for G20MM, G15MM, G10MM, G05MM using thin layer cold drying at 10°C.

*G10MM*. Figure 4a–d presents the comparison of predicted and experimental *MR* according to the moisture ratio for *G20MM*, *G15MM*, *G10MM*, *G05MM* using thin layer cold drying at 10°C.

### 3.2 The Evaluation of Cold Drying Models of Mackerel

The best similar mathematical thin layer equation between the data obtained from the cold drying experiments of horse mackerel was determined and calculated. The cold drying application included constant rate and decreasing rate periods. The modeling results obtained for *G20MM*, *G15MM*, *G10MM*, *G05MM* supported by 10°C cold drying are given in Tables 2, 3, 4, 5. After the values obtained during the cold air and thin layer drying of horse mackerel were modeled with 23 different models, the obtained values were evaluated with 13 different statistical evaluation criteria. In the review study conducted by Kucuk, Kilic, et al. (2014) and Kucuk, Midilli, et al. (2014), the most common 23 mathematical models and the statistical criteria determined to be widely used in the evaluation of these models in the study on mathematical models applied in thin layer drying and statistical criteria applied in the evaluation of these models were applied in low temperature drying technology and evaluated comprehensively. The criteria and data used to evaluate the mathematical models to which these statistical criteria were applied are given in detail in Tables 2, 3, 4, 5.

**TABLE 2:** Evaluation results for cold drying of the Mackerel for G20MM.

No	Model name	Evaluation criteria												
		RMSE	Residuals	EF	SEE	RSSE	MBE	*P*	*r*	RSS	SSE	*R* ^2^	*χ* ^2^	${\bar{R}}^{2}$
1.	Newton Lewis	0.0375	−0.1454	0.9897	0.0014	0.0014	0.0027	0.1824	0.9944	0.0744	0.0744	0.9897	0.0014	0.9895
2.	Page	0.0203	−0.1883	0.9970	0.0004	0.0004	0.0036	0.1155	0.9965	0.0217	0.0217	0.9970	0.0004	0.9969
3.	Modified Page	0.0203	−0.1941	0.9970	0.0004	0.0004	0.0037	0.1156	0.9965	0.0217	0.0217	0.9970	0.0004	0.9969
4.	Modified Page‐I	0.0203	−0.1982	0.9970	0.0004	0.0004	0.0037	0.1156	0.9965	0.0217	0.0217	0.9970	0.0004	0.9969
5.	Modified Page‐II	0.0375	−0.1449	0.9897	0.0015	0.0014	0.0027	0.1824	0.9944	0.0744	0.0744	0.9897	0.0015	0.9891
6.	Henderson & Pabis	0.0241	−0.0059	0.9957	0.0006	0.0006	0.0001	0.1206	0.9949	0.0309	0.0309	0.9957	0.0006	0.9956
7.	Logarithmic	0.0244	−0.0228	0.9956	0.0006	0.0006	0.0004	0.1339	0.9948	0.0315	0.0315	0.9956	0.0006	0.9954
8.	Midilli‐ Kucuk	0.0110	−0.0279	0.9991	0.0001	0.0001	0.0005	0.0620	0.9990	0.0064	0.0064	0.9991	0.0001	0.9990
9.	Demir et al.	0.0142	−0.0261	0.9985	0.0002	0.0002	0.0005	0.0811	0.9982	0.0107	0.0107	0.9985	0.0002	0.9984
10.	Two‐Term	0.0231	−0.0993	0.9961	0.0006	0.0005	0.0019	0.1323	0.9954	0.0284	0.0284	0.9961	0.0006	0.9958
11.	Two‐Term Exponential	0.0195	−0.1460	0.9972	0.0004	0.0004	0.0028	0.1087	0.9974	0.0201	0.0201	0.9972	0.0004	0.9971
12.	Verma et al.	0.0146	−0.1306	0.9984	0.0002	0.0002	0.0025	0.0814	0.9983	0.0112	0.0112	0.9984	0.0002	0.9984
13.	Approximation Diff.	0.0146	−0.1165	0.9984	0.0002	0.0002	0.0022	0.0815	0.9982	0.0114	0.0114	0.9984	0.0002	0.9983
14.	Modified Henderson	0.0229	−0.0796	0.9962	0.0006	0.0005	0.0015	0.1299	0.9954	0.0278	0.0278	0.9962	0.0006	0.9957
15.	Thompson	0.0262	−0.2650	0.9950	0.0007	0.0007	0.0050	0.1499	0.9943	0.0363	0.0363	0.9950	0.0007	0.9948
16.	Wang and Singh	0.0724	−0.5450	0.9616	0.0054	0.0052	0.0103	0.4095	0.9737	0.2779	0.2779	0.9616	0.0054	0.9601
17.	Hii et al.	0.0196	−0.1297	0.9972	0.0004	0.0004	0.0024	0.1140	0.9968	0.0203	0.0203	0.9972	0.0004	0.9969
18.	Simplified Fick diff.	0.0241	0.0191	0.9957	0.0006	0.0006	0.0004	0.1208	0.9949	0.0309	0.0309	0.9957	0.0006	0.9955
19.	Weibull	0.0203	−0.1890	0.9970	0.0004	0.0004	0.0036	0.1156	0.9965	0.0217	0.0217	0.9970	0.0004	0.9969
20.	Aghbashlo et al.	0.0288	−0.3092	0.9939	0.0009	0.0008	0.0058	0.1621	0.9939	0.0440	0.0440	0.9939	0.0009	0.9937
21.	Parabolic	0.0502	0.0112	0.9815	0.0027	0.0025	0.0002	0.1753	0.9805	0.1337	0.1337	0.9815	0.0027	0.9804
22.	Balbay and Şahin	0.0149	−0.0230	0.9984	0.0002	0.0002	0.0004	0.0836	0.9981	0.0118	0.0118	0.9984	0.0002	0.9982
23.	Alibas	0.0825	−0.0162	0.9502	0.0074	0.0068	0.0003	0.4009	0.9393	0.3606	0.3606	0.9502	0.0074	0.9460

In the obtained data, the error (residual) was selected based on the lowest values of *SSE*, *RMSE*, *χ* ^2^, residuals, *RSS*, *SEE*, *RSSE*, *MBE*, and *P*, while the highest values of *EF*, *r*, *R* ^2^ were accepted as the decisive criteria in the decision for the best mathematical model in the thin layer cold drying of mackerel. Table 2 gives the evaluation results for cold drying of the mackerel for *G20MM*.

In the analyses performed, as given in Tables 2, 3, 4, 5, the best common model in each experimental product was Balbay and Şahin, while the model showing the least fit was generally Wang and Singh, although it varied (Tables 2, 3, 4, 5). In the common model Balbay and Şahin, the *RMSE*, *r*, *R* ^2^, *χ* ^2^ values determined for G20MM were 0.0149, 0.9981, 0.9984, 0.0001, respectively, while the least fit model was determined as 0.0502, 0.9990, 0.9991, 0.0002. These results give good agreement with Akpinar (2006) and Kilic (2017).

Similarly, when the table is examined, when *SSE*, *RMSE*, *χ* ^2^, residuals, *RSS*, *SEE*, *RSSE*, *MBE* and *P* values are accepted as the lowest for Mackerel and *EF*, *r*, *R* ^2^ and adjusted ${\bar{R}}^{2}$ values are accepted as the highest, it will be seen that the best models for G20MM are Newton Lewis, Midilli‐Kucuk, Balbay and Şahin, Page model.

The most suitable model describing cold air‐drying of thin layer drying behavior of mackerel was observed as the highest value of the coefficient of determination (*R* ^2^), as the smallest value of *RMSE*, and the reduced chi‐square (*χ* ^2^). Table 3 gives the evaluation results for the cold drying of mackerel for G15MM.

**TABLE 3:** Evaluation results for cold drying of the Mackerel for G15MM.

No	Model name	Evaluation Criteria												
		RMSE	Residuals	EF	SEE	RSSE	MBE	*P*	*r*	RSS	SSE	*R* ^2^	*χ* ^2^	${\bar{R}}^{2}$
1.	Newton Lewis	0.0165	−0.0049	0.9981	0.0003	0.0003	0.0001	0.0928	0.9992	0.0129	0.0129	0.9981	0.0003	0.9981
2.	Page	0.0097	−0.0638	0.9993	0.0001	0.0001	0.0014	0.0576	0.9993	0.0044	0.0044	0.9993	0.0001	0.9993
3.	Modified Page	0.0095	−0.0566	0.9994	0.0001	0.0001	0.0012	0.0570	0.9993	0.0043	0.0043	0.9994	0.0001	0.9994
4.	Modified Page‐I	0.0096	−0.0372	0.9994	0.0001	0.0001	0.0008	0.0575	0.9993	0.0043	0.0043	0.9994	0.0001	0.9994
5.	Modified Page‐II	0.0165	−0.0160	0.9981	0.0003	0.0003	0.0003	0.0926	0.9992	0.0129	0.0129	0.9981	0.0003	0.9981
6.	Henderson & Pabis	0.0110	0.0480	0.9992	0.0001	0.0001	0.0010	0.0602	0.9991	0.0057	0.0057	0.9992	0.0001	0.9992
7.	Logarithmic	0.0106	−0.0128	0.9992	0.0001	0.0001	0.0003	0.0636	0.9991	0.0053	0.0053	0.9992	0.0001	0.9992
8.	Midilli‐ Kucuk	0.0164	0.0494	0.9981	0.0003	0.0003	0.0011	0.0922	0.9979	0.0126	0.0126	0.9981	0.0003	0.9981
9.	Demir et al.	0.0120	0.0207	0.9990	0.0002	0.0001	0.0004	0.0689	0.9989	0.0068	0.0068	0.9990	0.0002	0.9990
10.	Two‐Term	0.0101	0.0097	0.9993	0.0001	0.0001	0.0002	0.0577	0.9992	0.0048	0.0048	0.9993	0.0001	0.9993
11.	Two‐Term Exponential	0.0164	−0.0949	0.9981	0.0003	0.0003	0.0020	0.1024	0.9984	0.0126	0.0126	0.9981	0.0003	0.9981
12.	Verma et al.	0.0078	−0.0069	0.9996	0.0001	0.0001	0.0001	0.0442	0.9995	0.0029	0.0029	0.9996	0.0001	0.9996
13.	Approximation Diff.	0.0122	−0.0954	0.9990	0.0002	0.0001	0.0020	0.0713	0.9990	0.0070	0.0070	0.9990	0.0002	0.9990
14.	Modified Henderson	0.0103	0.0328	0.9993	0.0001	0.0001	0.0007	0.0576	0.9992	0.0050	0.0050	0.9993	0.0001	0.9993
15.	Thompson	0.0121	−0.0929	0.9990	0.0002	0.0001	0.0020	0.0709	0.9990	0.0068	0.0068	0.9990	0.0002	0.9990
16.	Wang and Singh	0.0482	−0.5391	0.9837	0.0024	0.0023	0.0115	0.2941	0.9937	0.1093	0.1093	0.9837	0.0024	0.9837
17.	Hii et al.	0.0087	−0.0166	0.9995	0.0001	0.0001	0.0004	0.0526	0.9994	0.0035	0.0035	0.9995	0.0001	0.9995
18.	Simplified Fick diff.	0.0110	0.0580	0.9992	0.0001	0.0001	0.0012	0.0605	0.9991	0.0057	0.0057	0.9992	0.0001	0.9992
19.	Weibull	0.0095	−0.0531	0.9994	0.0001	0.0001	0.0011	0.0571	0.9993	0.0043	0.0043	0.9994	0.0001	0.9994
20.	Aghbashlo et al.	0.0124	−0.1096	0.9989	0.0002	0.0002	0.0023	0.0738	0.9989	0.0073	0.0073	0.9989	0.0002	0.9989
21.	Parabolic	0.0281	−0.0061	0.9945	0.0008	0.0008	0.0001	0.1310	0.9941	0.0371	0.0371	0.9945	0.0008	0.9945
22.	Balbay and Şahin	0.0113	−0.0215	0.9991	0.0001	0.0001	0.0005	0.0634	0.9990	0.0060	0.0060	0.9991	0.0001	0.9991
23.	Alibas	0.0136	0.0231	0.9987	0.0002	0.0002	0.0005	0.0781	0.9986	0.0087	0.0087	0.9987	0.0002	0.9987

In the common model Balbay and Şahin, the *RMSE, r, R* ^2^, *χ* ^2^ values determined for G15MM were 0.0113, 0.9990, 0.999, 0.0001, respectively, while the least fit model was 0.0482, 0.9937, 0.9837, 0.0024. Similarly, when the table is examined, when *SSE, RMSE, χ* ^2^, residuals, *RSS, SEE, RSSE, MBE* and *p* values are accepted as the lowest for Mackerel. In addition, *EF*, *r*, *R* ^2^ and adjusted ${\bar{R}}^{2}$ values are accepted as the highest. It will be seen that the best models for G15MM, the most suitable 7 model can be selected as Henderson & Pabis, Logarithmic (Asymptotic), Two‐Term, Verma et al., Modified Henderson, Simplified Fick diff., Balbay and Şahin model. Table 4 the evaluation results for cold drying of the Mackerel for G10MM.

**TABLE 4:** Evaluation results for cold drying of the mackerel for G10MM.

No	Model name	Evaluation criteria												
		RMSE	Residuals	EF	SEE	RSSE	MBE	*P*	*r*	RSS	SSE	*R* ^2^	*χ* ^2^	${\bar{R}}^{2}$
1.	Newton Lewis	0.0191	0.1484	0.9974	0.0004	0.0004	0.0035	0.1095	0.9988	0.0156	0.0156	0.9974	0.0004	0.9974
2.	Page	0.0129	0.0591	0.9988	0.0002	0.0002	0.0014	0.0721	0.9988	0.0071	0.0071	0.9988	0.0002	0.9988
3.	Modified Page	0.0127	0.0656	0.9989	0.0002	0.0002	0.0015	0.0744	0.9988	0.0069	0.0069	0.9989	0.0002	0.9988
4.	Modified Page‐I	0.0127	0.0656	0.9989	0.0002	0.0002	0.0015	0.0744	0.9988	0.0069	0.0069	0.9989	0.0002	0.9988
5.	Modified Page‐II	0.0191	0.1350	0.9974	0.0004	0.0004	0.0031	0.1095	0.9987	0.0156	0.0156	0.9974	0.0004	0.9972
6.	Henderson & Pabis	0.0163	0.1882	0.9981	0.0003	0.0003	0.0044	0.0861	0.9983	0.0114	0.0114	0.9981	0.0003	0.9980
7.	Logarithmic	0.0100	−0.0150	0.9993	0.0001	0.0001	0.0003	0.0683	0.9992	0.0043	0.0043	0.9993	0.0001	0.9992
8.	Midilli‐ Kucuk	0.0149	0.0451	0.9984	0.0002	0.0002	0.0010	0.0972	0.9983	0.0095	0.0095	0.9984	0.0002	0.9983
9.	Demir et al.	0.0109	−0.0158	0.9992	0.0001	0.0001	0.0004	0.0697	0.9991	0.0051	0.0051	0.9992	0.0001	0.9991
10.	Two‐Term	0.0129	0.0627	0.9988	0.0002	0.0002	0.0015	0.0770	0.9988	0.0071	0.0071	0.9988	0.0002	0.9987
11.	Two‐Term Exponential	0.0138	0.0021	0.9987	0.0002	0.0002	0.0000	0.0956	0.9986	0.0082	0.0082	0.9987	0.0002	0.9986
12.	Verma et al.	0.0113	−0.0113	0.9991	0.0001	0.0001	0.0003	0.0759	0.9990	0.0055	0.0055	0.9991	0.0001	0.9990
13.	Approximation Diff.	0.0124	0.0155	0.9989	0.0002	0.0002	0.0004	0.0820	0.9988	0.0066	0.0066	0.9989	0.0002	0.9988
14.	Modified Henderson	0.0119	0.0228	0.9990	0.0002	0.0001	0.0005	0.0775	0.9989	0.0060	0.0060	0.9990	0.0002	0.9988
15.	Thompson	0.0120	0.0144	0.9990	0.0002	0.0001	0.0003	0.0782	0.9989	0.0062	0.0062	0.9990	0.0002	0.9989
16.	Wang and Singh	0.0457	−0.3551	0.9853	0.0022	0.0021	0.0083	0.3106	0.9909	0.0897	0.0897	0.9853	0.0022	0.9846
17.	Hii et al.	0.0126	0.0518	0.9989	0.0002	0.0002	0.0012	0.0756	0.9988	0.0069	0.0069	0.9989	0.0002	0.9987
18.	Simplified Fick diff.	0.0163	0.1814	0.9981	0.0003	0.0003	0.0042	0.0865	0.9982	0.0114	0.0114	0.9981	0.0003	0.9980
19.	Weibull	0.0127	0.0694	0.9989	0.0002	0.0002	0.0016	0.0743	0.9988	0.0069	0.0069	0.9989	0.0002	0.9988
20.	Aghbashlo et al.	0.0119	−0.0107	0.9990	0.0001	0.0001	0.0002	0.0767	0.9990	0.0061	0.0061	0.9990	0.0001	0.9990
21.	Parabolic	0.0297	0.0118	0.9938	0.0010	0.0009	0.0003	0.1892	0.9931	0.0380	0.0380	0.9938	0.0010	0.9933
22.	Balbay and Şahin	0.0100	−0.0145	0.9993	0.0001	0.0001	0.0003	0.0677	0.9992	0.0043	0.0043	0.9993	0.0001	0.9992
23.	Alibas	0.0119	0.0187	0.9990	0.0002	0.0001	0.0004	0.0787	0.9989	0.0061	0.0061	0.9990	0.0002	0.9989

The *RMSE, r, R* ^2^, *χ* ^2^ values determined for G10MM were 0.0100, 0.9992, 0.9993, 0.0001 in the common model Balbay and Şahin, respectively, while the least fit model was 0.0457, 0.9909, 0.9853, 0.0022 as Akpinar (2006).

Similarly, the most suitable 7‐model can be selected as Demir et al., Logarithmic (Asymptotic), Two‐Term, Thompson, Verma et al., Balbay, and Şahin and Alibas model for G10MM. Table 5 gives the evaluation results for cold drying of the Mackerel as G05MM.

**TABLE 5:** Evaluation results for cold drying of the Mackerel for G05MM.

No	Model name	Evaluation criteria												
		RMSE	Residuals	EF	SEE	RSSE	MBE	*P*	*r*	RSS	SSE	*R* ^2^	*χ* ^2^	${\bar{R}}^{2}$
1.	Newton Lewis	0.0338	0.2702	0.9916	0.0012	0.0011	0.0093	0.3283	0.9956	0.0331	0.0331	0.9916	0.0012	0.9913
2.	Page	0.0240	0.1416	0.9958	0.0006	0.0006	0.0049	0.2368	0.9957	0.0167	0.0167	0.9958	0.0006	0.9954
3.	Modified Page	0.0240	0.1423	0.9958	0.0006	0.0006	0.0049	0.2387	0.9956	0.0167	0.0167	0.9958	0.0006	0.9954
4.	Modified Page‐I	0.0240	0.1423	0.9958	0.0006	0.0006	0.0049	0.2387	0.9956	0.0167	0.0167	0.9958	0.0006	0.9954
5.	Modified Page‐II	0.0338	0.2702	0.9916	0.0013	0.0011	0.0093	0.3283	0.9956	0.0331	0.0331	0.9916	0.0013	0.9906
6.	Henderson & Pabis	0.0314	0.2897	0.9927	0.0011	0.0010	0.0100	0.2898	0.9938	0.0286	0.0286	0.9927	0.0011	0.9922
7.	Logarithmic	0.0104	0.0145	0.9992	0.0001	0.0001	0.0005	0.1107	0.9991	0.0032	0.0032	0.9992	0.0001	0.9991
8.	Midilli‐ Kucuk	0.0078	0.0160	0.9996	0.0001	0.0001	0.0006	0.0785	0.9995	0.0018	0.0018	0.9996	0.0001	0.9995
9.	Demir et al.	0.0090	0.0067	0.9994	0.0001	0.0001	0.0002	0.0936	0.9994	0.0024	0.0024	0.9994	0.0001	0.9993
10.	Two‐Term	0.0114	−0.0092	0.9991	0.0001	0.0001	0.0003	0.1220	0.9990	0.0037	0.0037	0.9991	0.0001	0.9989
11.	Two‐Term Exponential	0.0282	0.2029	0.9941	0.0009	0.0008	0.0070	0.2795	0.9943	0.0231	0.0231	0.9941	0.0009	0.9937
12.	Verma et al.	0.0088	−0.0148	0.9994	0.0001	0.0001	0.0005	0.0904	0.9994	0.0022	0.0022	0.9994	0.0001	0.9994
13.	Approximation Diff.	0.0085	−0.0140	0.9995	0.0001	0.0001	0.0005	0.0861	0.9994	0.0021	0.0021	0.9995	0.0001	0.9994
14.	Modified Henderson	0.0098	−0.0134	0.9993	0.0001	0.0001	0.0005	0.1029	0.9992	0.0028	0.0028	0.9993	0.0001	0.9991
15.	Thompson	0.0199	0.0820	0.9971	0.0004	0.0004	0.0028	0.2016	0.9969	0.0115	0.0115	0.9971	0.0004	0.9969
16.	Wang and Singh	0.0545	−0.2441	0.9782	0.0032	0.0030	0.0084	0.5786	0.9859	0.0860	0.0860	0.9782	0.0032	0.9765
17.	Hii et al.	0.0066	0.0004	0.9997	0.0001	0.0000	0.0000	0.0615	0.9997	0.0013	0.0013	0.9997	0.0001	0.9996
18.	Simplified Fick diff.	0.0314	0.2989	0.9927	0.0011	0.0010	0.0103	0.2891	0.9940	0.0286	0.0286	0.9927	0.0011	0.9919
19.	Weibull	0.0240	0.1430	0.9958	0.0006	0.0006	0.0049	0.2387	0.9956	0.0167	0.0167	0.9958	0.0006	0.9954
20.	Aghbashlo et al.	0.0178	0.0507	0.9977	0.0003	0.0003	0.0017	0.1907	0.9976	0.0092	0.0092	0.9977	0.0003	0.9975
21.	Parabolic	0.0377	0.0003	0.9895	0.0016	0.0014	0.0000	0.3606	0.9889	0.0412	0.0412	0.9895	0.0016	0.9883
22.	Balbay and Şahin	0.0089	−0.0012	0.9994	0.0001	0.0001	0.0000	0.0906	0.9994	0.0023	0.0023	0.9994	0.0001	0.9993
23.	Alibas	0.0180	−0.0091	0.9976	0.0004	0.0003	0.0003	0.1926	0.9974	0.0094	0.0094	0.9976	0.0004	0.9971

In the common model Balbay and Şahin, the *RMSE*, *r*, *R* ^2^, *χ* ^2^ values determined for G05MM were 0.089, 0.9994, 0.9994, 0.0001, respectively, while the least fit model was 0.0545, 0.9940, 0.9782, 0.0013.

Finally, when the table is examined for *SSE*, *RMSE*, *χ* ^2^, residuals, *RSS*, *SEE*, *RSSE*, *MBE* and *P* values are accepted as the lowest for Mackerel. In addition, *EF*, *r*, *R* ^2^ and ${\bar{R}}^{2}$ values are accepted as the highest. It is found that the best models for G05MM, the most suitable 7 model can be selected as Logarithmic (Asymptotic), Demir et al., Two‐Term, Verma et al., Thompson, Balbay and Şahin and Alibas model. It is known that many factors connected to raw materials and drying parameters affect the choice of the most appropriate mathematical model (Baidhe and Clementson 2024; Bhattacharjee et al. 2024; Kilic 2009, 2022). On the other hand, although different mathematical models are applied in different products depending on experimental parameters, the statistical criteria applied in the comparison and selection of the most suitable model are standard. Therefore, the same brokers can be applied in the selection of the most suitable mathematical models required for drying all foods (Djekic et al. 2019; Baidhe and Clementson 2024; Kilic 2022; Akpinar 2006; Kucuk, Midilli, et al. 2014; Bhattacharjee et al. 2024).

## 4 Conclusions

In this study, common mathematical models and statistical evaluation criteria in the literature were evaluated comprehensively. In this context, horse mackerel ( *T. trachurus* ) fish samples with different sample thicknesses (G20MM, G15MM, G10MM, G05MM) were modeled using the environmentally friendly cold drying (LTHV) method recommended for sensitive functional foods with bioactive content and were examined comprehensively with the model and evaluation criteria in the literature. For this purpose, 23 semi‐theoretical and/or empirical mathematical equations common in food technology were used. Regression analysis was performed on the obtained data with 13 commonly used statistical evaluation criteria.

For the G20MM group, 4 mathematical models, and for each of the G15MM, G10MM, and G05MM groups, 7 mathematical models were determined as the most suitable models (Figures 3 and 4). Balbay and Şahin model was selected as the most appropriate mathematical model in all experimental groups. In the selection of the most suitable mathematical models, the most common 13 statistical evaluation criteria were applied, and it was concluded that using 4 evaluation criteria would be sufficient, based on the maximum values obtained from *r*, *R* ^2^ and the minimum values of *χ* ^2^, *RMSE*, which coincide with the literature information (Tables 2, 3, 4, 5). Since the drying results, where the cold drying parameters are the same, give similar results for each model, only one can be selected and the reduced chi‐square (*χ* ^2^) and standard error of estimate (SEE) values can be applied. It was concluded that SST was similar in all models for the same drying conditions and in this context, SST did not give good results as a distinguishing criterion in the evaluations obtained in the data and therefore should not be used.

**Nomenclature**
*t*
: drying time (h)
*T*
: drying temperature (°C)
*h*
: hour (h)
*v*
: cold air velocity constant, h^−1^
*wb*
: food wet basis (%)
*db*
: food dry basis (%)
*dai*
: the drying air inlet (m/s)
*dao*
: the air output of system (m/s)
constants: *a*, *b*, *c*, *g*, *h*
*k*, *k*_0_, *k*_1_, *k*_2_
: constant (h^−1^)
*o*
: observed
*p*
: predicted
*n*
: the drying constant number
*N*
: the drying observation number
*e*
: equilibrium
*aw*
: water activity
*LTHV*
: low temperature and high velocity drying
*SSE*
: sum square error
*CP*
: the system control point
*M*
: cold drying loss (g kg^−1^)
*M*_*i*_
: Mackerel sample weight (g) for *t* = 0
*M*_*e*_
: Mackerel sample equilibrium weight (g)
*M*_*o*_
: Mackerel sample weight (g) for *t* = 0
*M*_*t*_
: Mackerel sample weight (g) for *t = t*
*Rh*
: Relative humidity
*L*
: dimensionless drying constant
*MBE*
: mean bias error
*P*
: the relative percentage error of mea*n*
*r*
: the coefficient of correlation
*R* ^2^
: the determination coefficient
${\bar{R}}^{2}$
: the adjusted *R* ^2^
RSS: squares sum
RSSE: sum square error as reduced
SEE: estimated standard error
SST: total sum squares
*R*
: Regression
*exp*.: Experimental
*pre*
: Predicted
*RMSE*
: the square error of root mean
*χ* ^2^
: the chi‐square reduced of variance
*Z*
: the constant number
*W*
: moisture content (%)
*EF*
: the efficiency of data
*E*_*MD*_
: the relative percentage deviation of mean
Avg.: the average of data
*MR*
: the moisture ratio of sample
*MSR*
: Mass shrinkage of sample
*E*_*RMS*_
: the mean square error of roots
*E*
: the relative deviation of percent mean

## Author Contributions

**Aydin Kilic:** conceptualization (equal), data curation (equal), formal analysis (equal), funding acquisition (equal), investigation (equal), methodology (equal), project administration (equal), resources (equal), software (equal), supervision (equal), validation (equal), visualization (equal), writing – original draft (equal), writing – review and editing (equal).

## Conflicts of Interest

The author declares no conflicts of interest.

## Data Availability

Data openly available in a public repository that issues datasets with DOIs.
